# Plasma Levels of Neopterin and C-Reactive Protein (CRP) in Tuberculosis (TB) with and without HIV Coinfection in Relation to CD4 Cell Count

**DOI:** 10.1371/journal.pone.0144292

**Published:** 2015-12-02

**Authors:** Sten Skogmar, Thomas Schön, Taye Tolera Balcha, Erik Sturegård, Marianne Jansson, Per Björkman

**Affiliations:** 1 Infectious Disease Research Unit, Department of Clinical Sciences Malmö, Lund University, Malmö, Sweden; 2 Department of Medical Microbiology, Faculty of Health Sciences, Linköping University, Linköping, Sweden; 3 Department of Clinical Microbiology and Infectious diseases, Kalmar County Hospital, Kalmar, Sweden; 4 Ministry of Health, Addis Ababa, Ethiopia; 5 Medical Microbiology, Department of Laboratory Medicine Malmö, Lund University, Malmö, Sweden; 6 Department of Laboratory Medicine, Lund University, Lund, Sweden; 7 Department of Microbiology, Tumor and Cell biology, Karolinska Institute, Stockholm, Sweden; University of Pittsburgh Center for Vaccine Research, UNITED STATES

## Abstract

**Background:**

While the risk of TB is elevated in HIV-positive subjects with low CD4 cell counts, TB may in itself be associated with CD4 lymphocytopenia. We investigated markers of immune activation (neopterin) and inflammation (CRP) in TB patients with and without HIV coinfection and their association with CD4 cell levels, and determined their predictive capacity as alternative markers of advanced immunosuppression.

**Methods:**

Participants selected from a cohort of adults with TB at Ethiopian health centers (195 HIV+/TB+, 170 HIV-/TB+) and 31 controls were tested for plasma levels of neopterin and CRP. Baseline levels of neopterin and CRP were correlated to CD4 cell count before and after anti-TB treatment (ATT). The performance to predict CD4 cell strata for both markers were investigated using receiver operating curves.

**Results:**

Levels of both biomarkers were elevated in TB patients (neopterin: HIV+/TB+ 54 nmol/l, HIV-/TB+ 23 nmol/l, controls 3.8 nmol/l; CRP: HIV+/TB+ 36 μg/ml, HIV-/TB+ 33 μg/ml, controls 0.5 μg/ml). Neopterin levels were inversely correlated (-0.53, p<0.001) to CD4 cell count, whereas this correlation was weaker for CRP (-0.25, p<0.001). Neither of the markers had adequate predictive value for identification of subjects with CD4 cell count <100 cells/mm^3^ (area under the curve [AUC] 0.64 for neopterin, AUC 0.59 for CRP).

**Conclusion:**

Neopterin levels were high in adults with TB, both with and without HIV coinfection, with inverse correlation to CD4 cell count. This suggests that immune activation may be involved in TB-related CD4 lymphocytopenia. However, neither neopterin nor CRP showed promise as alternative tests for immunosuppression in patients coinfected with HIV and TB.

## Introduction

TB is the most common opportunistic infection (OI) and cause of death in people living with HIV (PLHIV) globally, with the highest case burden in sub-Saharan Africa [1]. In HIV-positive persons the risk of active TB is inversely correlated to CD4 cell levels [2]. Although CD4 cell depletion is characteristic of HIV disease, subnormal CD4 cell levels can occur in other conditions [3], which may coexist in PLHIV. This includes active TB [4–6]; however the mechanisms involved in TB-related CD4 lymphocytopenia are unclear.

In HIV infection, the main cause of CD4 cell depletion and disease progression is chronic immune activation [7,8]. Low-grade chronic immune activation is mainly caused by bacterial translocation from the gastrointestinal tract [9]. However, it is also possible that OI:s could contribute to immune activation (IA), thus creating a vicious spiral in HIV-infected subjects with pre-existent immunosuppression [10]. A central component in the pathogenesis of TB is the activation of macrophages by T-cells. We hypothesized that IA may be involved in CD4 cell lymphocytopenia also in HIV-negative individuals with TB.

We have recently reported a relationship between CD4 cell levels and disease severity in a cohort of Ethiopian TB patients with and without HIV coinfection [4]. In the present study, we aimed to investigate IA in TB-related CD4 lymphocytopenia by determining plasma levels of neopterin and CRP (reflecting immune activation and systemic inflammation, respectively) in cohort participants in relation to CD4 cell count before and after anti-TB treatment. In addition, we aimed to investigate the potential use of these plasma markers as alternative tests for assessment of HIV-related immunosuppression in TB/HIV coinfection.

## Methods

### Study participants

Participants were selected and retrospectively analyzed from a prospective cohort study encompassing 1116 TB patients (307 HIV+, 809 HIV-negative; described in detail previously), with the overall aim to investigate immunosuppression in TB with and without HIV coinfection [4,11].

Patients were recruited and followed up at eight outpatient TB clinics (based in 6 health centers, 1 regional hospital and 1 zonal hospital) in the Oromia region, Ethiopia, between September 2010 and September 2012. Inclusion criteria were: diagnosis of active TB, age 18 years or greater, residence in the clinic uptake area, and consent to HIV testing. Subjects having received ATT for more than 2 weeks for their current episode of TB, or who had been treated for TB within the preceding 6 months were excluded, as were persons with current or previous antiretroviral therapy (ART). A control group of healthy individuals was recruited at a voluntary HIV counseling and testing clinic located at one of the study health centers. HIV-negative subjects without signs or symptoms suggestive of TB or other illness were eligible as controls.

TB patients were followed during the course of ATT, with clinical evaluation and CD4 cell analysis at initiation, and at 2 and 6 months of ATT. Active TB was defined according to Ethiopian national guidelines [12]. Pulmonary TB was diagnosed in subjects with at least one positive sputum smear for acid-fast bacilli, or in sputum smear-negative patients with compatible clinical presentation and chest radiography, and lack of improvement after antibiotic therapy. Peripheral TB lymphadenitis was diagnosed with fine needle aspiration cytology; other forms of extrapulmonary TB were diagnosed using targeted investigations depending on disease manifestation.

For all participants, HIV testing was done according to national guidelines by two rapid tests [13]. ART was prescribed to HIV+ patients according to Ethiopian guidelines, (at the time of data collection: CD4 cell count <350 cells/mm^3^ for pulmonary TB and irrespective of CD4 cell count for extrapulmonary TB) [13].

**Fig 1:**
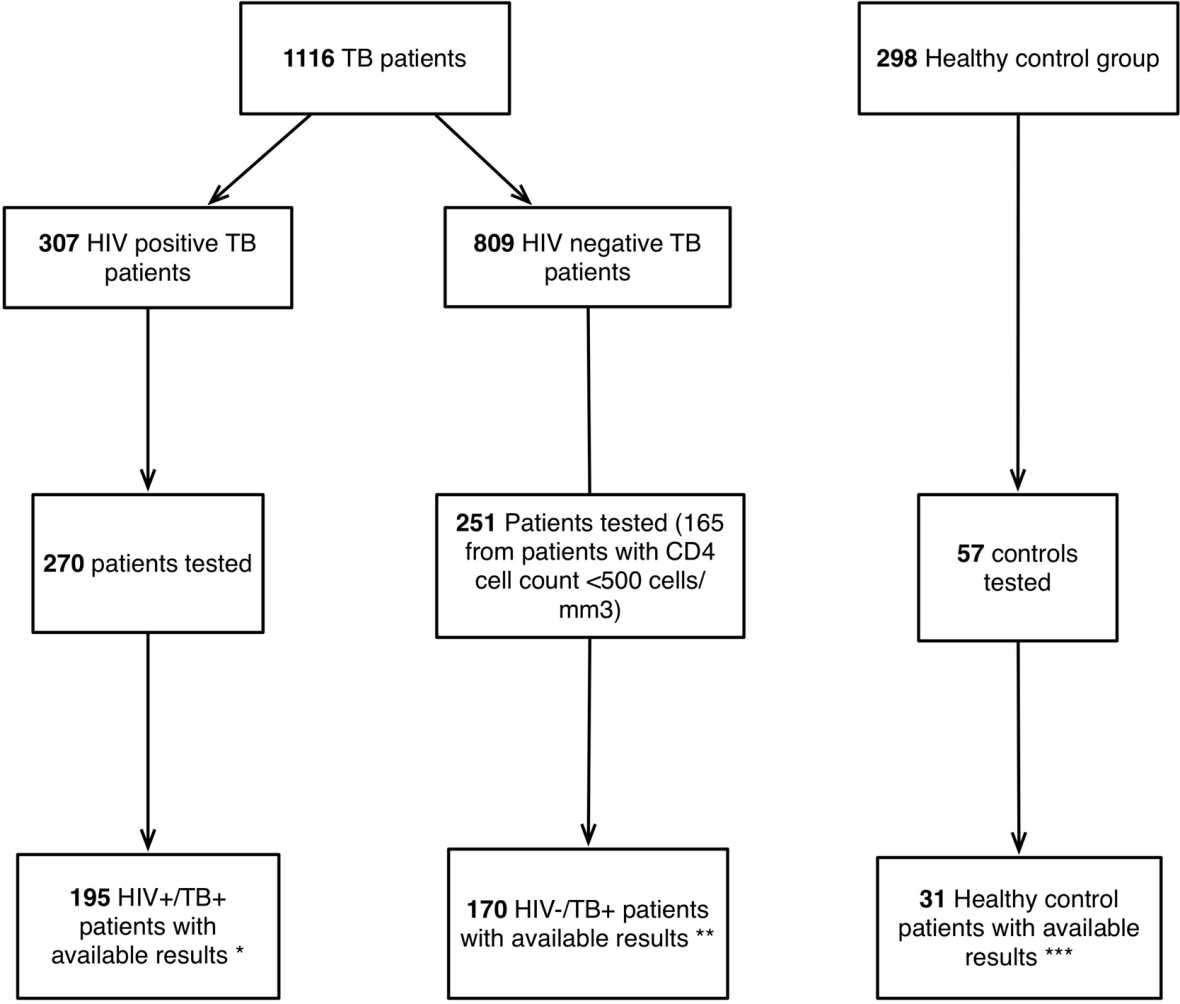
Flow chart of patients included in the study. Only patients that met selection and analysis criteria were included in the analysis (195 HIV+/TB+; 170 HIV-/TB+; 31 healthy controls). * 37 subject samples not available for testing, 75 subjects did not fulfil criteria for test result. ** 81 subjects did not fulfil criteria for test result. *** 26 subjects did not fulfil criteria for test result.

For the current study, participants were selected from the original cohort and stored baseline samples were analyzed based on HIV serostatus and CD4 cell levels (as detailed in Fig 1): 1) all HIV/TB co-infected patients (HIV+/TB+); 2) all HIV-/TB+ patients with baseline CD4 cell levels <500 cells/mm^3^; 3) a subset of consecutive HIV-/TB+ patients with baseline CD4 cell levels >500 cells/mm^3^ 4) a subset of HIV-/TB- controls.

All subjects provided written informed consent to participate in the study. The study was approved by the National Ethics Review Committee at the Ministry of Science and Technology of Ethiopia and by the Ethical Review Board at Lund University, Sweden.

### Laboratory methods

At baseline, 2 month and 6 month visits, 8 ml venous blood was collected in EDTA-tubes for determination of CD4 cell counts by flow cytometry, using standard protocols (using either FACSCount or FACSCalibur [BD Biosystems, San Jose California]). Remaining plasma was aliquoted and stored at -80°C.

Plasma aliquots were analyzed at the end of cohort inclusion, using ELISA kits for neopterin and highly sensitive CRP (IBL international, Hamburg, Germany) following the manufacturer’s instructions. Assays were performed in duplicates with three kit-independent controls included in each separate run, representing high, medium and low values. The optical density (OD) readings were transposed to levels of neopterin and CRP from a standard curve from a 4-spline nonparametric reader fit algorithm as recommended (Miraibio group, Hitachi solutions, San Bruno, CA, USA). Mean values of duplicate samples from each individual were used. The kit-independent controls were used to assess the inter-assay variation. A 10% difference in kit-independent controls as well as between duplicates was accepted according to the manufacturer’s recommendations. Patients with greater variation in these results were excluded from further analysis. All laboratory analyses were performed at the Adama Regional Laboratory, Ethiopia.

### Statistical analysis

The study sample of HIV+/TB+ subjects was subdivided according to commonly used CD4 cell strata; <100 cells/mm^3^, 100–350 cells/mm^3^, >350 cells/mm^3^. HIV-/TB+ patients were subdivided into groups with low (<500 cells/mm^3^) and normal (≥500 cells/mm^3^) CD4 cell count. For analysis of CD4 cell evolution during ATT, we classified subjects in two categories: “increasing” CD4 cell count (follow-up value increased with at least 50 cells/mm^3^ as compared to baseline count) and “no increase” in CD4 cell count (follow up value not increased with at least 50 cells/mm^3^ or decreased as compared to baseline count). For this analysis, only patients with follow up CD4 cell count after 6 months were included. TB+/HIV+ subjects who initiated ART during this period were also excluded from analysis of CD4 cell evolution.

Neopterin and CRP data were analyzed with nonparametric statistical tests and divided into medians and quartiles. An upper cut-off level for neopterin of 111 nmol/l was used according to the manufacturer’s instruction. Values for neopterin <10nmol/l and CRP<8μg/ml were considered to be normal. Differences between groups of median biomarker concentrations were tested with Mann Whitney U test and correlations were analyzed with the Spearman rank (SR) test. Receiver operating curves (ROC) and areas under the curves (AUC) were calculated to evaluate biomarker predictive performance (where an AUC of 1.0 is perfect discriminatory capability and 0.5 is chance [14]) for the aforementioned CD4 cell count cut-off thresholds. All statistical analyses were performed using SPSS v.20 (IBM).

## Results

### Participant characteristics

For this study, 365 patients and 31 controls were included (195 HIV+/TB+ and 170 HIV-/TB+) from the original cohort. (Fig 1). There was no difference between the subset of HIV+/TB+ patients that was tested for biomarkers compared to HIV+/TB+ patients who were not tested with regard to gender, TB manifestations, mid upper arm circumference (MUAC) and body mass index (BMI); however, persons not included had non-significantly higher median CD4 cell count (192 vs. 150 cells/mm^3^, p = 0.102). HIV-negative patients included for testing were also similar to non-included HIV-negative patients; however, due to the study design these subjects were more likely to be smear positive (44% vs. 36%) and had significantly lower CD4 cell count (438 vs 715 cells/mm^3^; p = 0.001; Table 1).

**Table 1:** Baseline characteristics of included and non-included TB-patients with or without HIV ^a^ and healthy controls ^b^.

	HIV+/TB patients		HIV-/TB patients ^c^		Healthy controls (n = 31)
	Included (n = 195)	Not included (n = 112)	Included (n = 170)	Not included (n = 639)	
**Age, median (IQR)**	32 (28–40)	33 (28–40)	30 (23–44)	28 (22–42)	22 (20–25)
**Female gender, n (%)**	99 (51)	52 (46)	67 (39)	310 (49)	22 (71)
**Smear positive pulmonary TB, n (%)**	62 (32)	37 (33)	75 (44)	231 (36)	-
**Smear negative pulmonary TB, n (%)**	62 (32)	32 (29)	43 (35)	173 (27)	-
**Extrapulmonary TB, n (%)** ^d^	74 (38)	49 (44)	54 (32)	244 (38)	-
**BMI kg/m** ^**2**^ **(IQR)** ^e^	17.5 (16–19.5)	17.6 (16.1–19.5)	18 (16.4–19.7)	18.2 (16.5–20)	21.6 (20.7–22.8)
**MUAC cm (IQR)** ^f^	21 (19–22)	20 (19–22.4)	21 (20–23)	22 (20–23)	24.5 (24–26.5)
**CD4 count (cells/mm** ^**3**^ **; median [IQR])**	192 (106–344)	150 (82–310)	438 (323–688)	715 (569–925)	1022 (869–1118)

^a^ Total number of recruited subjects with active TB in the cohort was 1116

^b^ Total number of recruited healthy controls in the cohort was 298

c All patients with CD4<500 cells/mm^3^ were selected for inclusion. For patients with CD4≥500cells/mm^3^ a consecutive number of patients were selected for inclusion.

^d^ Patients could have a simultaneous diagnosis of extrapulmonary and pulmonary TB (included HIV+ patients, n = 3, included HIV-negative patients, n = 2).

^e^ BMI is the calculated Body mass index (weight/height^2^).

^f^ MUAC is the mid upper arm circumference measured by a measuring tape.

### Plasma levels of neopterin and CRP in TB with and without HIV coinfection

To study the degree of immune activation in TB patients with or without HIV coinfection plasma levels of neopterin and CRP were determined.

The median level of neopterin in TB patients was higher (median 37.3 nmol/l, IQR 18.8–78.3) than in the control group (median 3.8 nmol/, IQR 1.5–5.2; p<0.001; Table 2). Median neopterin levels were significantly higher in HIV+/TB+ patients (median 54 nmol/l, IQR 32–108) than in HIV-/TB+ patients (median 23 nmol/l, IQR 13–45; p<0.001). Similarly, CRP levels were increased in TB patients compared to controls (36 μg/ml [IQR 12–74] vs. 0.5 μg/ml [0.2–1.2]; p<0.001), but no significant difference was observed when comparing CRP levels in HIV+ and HIV-negative patients (median 35 μg/ml [IQR 13–73] vs. 31 μg/ml [IQR 11–70], respectively; p = 0.613). The control group had median neopterin (3.8 nmol/l [IQR 1.6–5.5] and CRP levels (0.5 μg/ml [IQR 0.2–1.2] within the normal range.

**Table 2:** Neopterin and CRPlevels in different CD4 cell count strata in 365 TB patients with and without HIV and 31 controls.

**CD4 cell count strata**	**Neopterin in HIV+/TB patients** ^a^	**Neopterin in HIV-/TB patients** ^a^	**Neopterin in Healthy controls** ^a^
All	54 (30–111) (n = 195)	23.2 (14–44) (n = 170)	3.8 (1.6–5.5) (n = 31)
>500 cells/mm^3^	32 (22–50) (n = 22)	14 (10–22) (n = 67)	-
>350 cells/mm^3^	36 (24–56) (n = 48)	20 (12–38) (n = 113)	-
100–350 cells/mm^3^	63 (35–111) (n = 103)	30 (17–51) (n = 54)	-
<100 cells/mm^3^	77 (43–111) (n = 44)	82 (79–104) (n = 3)	-
**CD4 cell count strata**	**CRP in HIV+/TB patients** ^b^	**CRP in HIV-/TB patients** ^b^	**CRP in Healthy controls** ^b^
All	36 (12–74) (n = 195)	33.4 (12–79) (n = 170)	0.5 (0.2–1.2) (n = 31)
>500 cells/mm^3^	16 (2.8–41) (n = 22)	19 (6,3–53) (n = 67)	-
>350 cells/mm^3^	17 (5.3–51) (n = 48)	27 (10–66) (n = 113)	-
100–350 cells/mm^3^	40 (16–75) (n = 103)	56 (21–90) (n = 54)	-
<100 cells/mm^3^	47 (13–95) (n = 44)	33 (33–33) (n = 3)	-

^a^ Neopterin levels (nmol/l), median (IQR) and number of study subject (n =)

^b^ CRP levels (μg/ml), Median (IQR) and number of study subject (n =)

### Correlation between CD4 cell count and plasma levels of neopterin and CRP

In the analysis of potential associations between immune activation and CD4 lymphocytopenia we observed that plasma neopterin levels showed a significant inverse correlation to CD4 cell count for both HIV+/TB patients (SR rho -0.35, p<0.001) and for HIV-/TB patients (SR rho -0.51, p<0.001), see S1 Fig. Significantly higher levels were noted with lower CD4 cell count strata in TB cases, both with and without HIV coinfection. The median neopterin concentration in HIV+/TB+ patients with CD4 cell count <100 cells/mm^3^ was 66 nmol/l (IQR 42–111) compared to 35 nmol/l (24–55) in patients with CD4 cell count >350 cells/mm^3^; p<0.001). Similarly, CRP had an inverse correlation to CD4 cell count, but weaker than for neopterin (SR rho -0.25, p<0.001). The highest levels of CRP were found in the intermediate CD4 cell count strata (100–350 cells/mm^3^: 56 μg/ml vs. 27 μg/ml for CD4 cell count>350 cells/mm^3^ and 33 μg/ml for CD4 cell count <100 cells/mm^3^). Neopterin and CRP levels in relation to CD4 cell strata are presented in Table 2.

### Baseline levels of neopterin and CD4 cell count evolution during ATT

**Fig 2:**
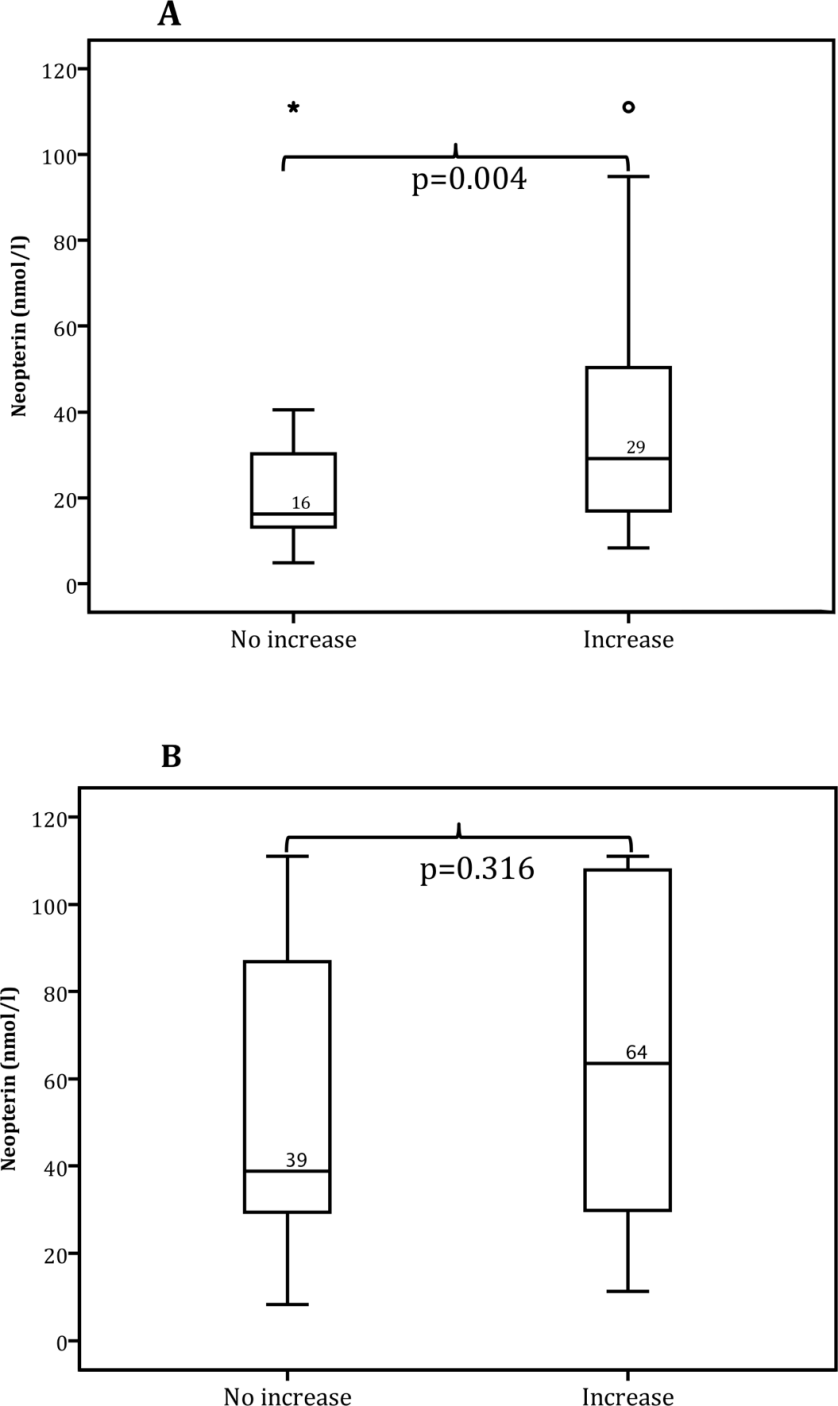
Baseline neopterin in relation to evolution of CD4 cell count at completion of ATT subdivided into two groups; No increase (CD4 cell count ≤50 cells/mm^3^ or unchanged CD4 cell count, ± 50 cells/mm^3^ after 6 months ATT), or increase (CD4 ≥50 cells/mm^3^ after 6 months ATT). Graph **A** displays **HIV-**patients (n = 85 of whom 58 had an increase in CD4 cell count). Baseline median CD4 cell count for those with increasing CD4 cell count was 405 cells/mm^3^ and 496 cells/mm^3^ for those without increasing CD4 cell count (n = 27). Graph **B** displays **HIV+** patients who did not start ART (n = 43 of whom 22 had an increase in CD4 cell count). Baseline median CD4 cell count for those with increasing CD4 cell count was 255 cells/mm^3^ and 411 cells/mm^3^ for those without increasing CD4 cell count (n = 21).

With the aim to analyze the relation between immune activation and evolution of CD4 cell count during ATT, baseline neopterin levels were correlated to CD4 count evolution during follow-up. Neopterin levels showed a positive correlation with CD4 cell count at 6 months (SR rho 0.34), p<0.001). HIV-/TB+ patients (n = 88) with increasing CD4 cell count (n = 58) had significantly higher neopterin levels compared to those with unchanged CD4 count (n = 30; 29nmol/l vs. 16nmol/l, p = 0.004). In HIV+/TB+ patients (n = 43) neopterin levels were also higher in patients with increasing CD4 cell counts during ATT (64 nmol/l vs. 39nmol/l), but this difference was non-significant (p = 0.316), (Fig 2).

### Neopterin and CRP as predictive markers of CD4 cell count

**Fig 3:**
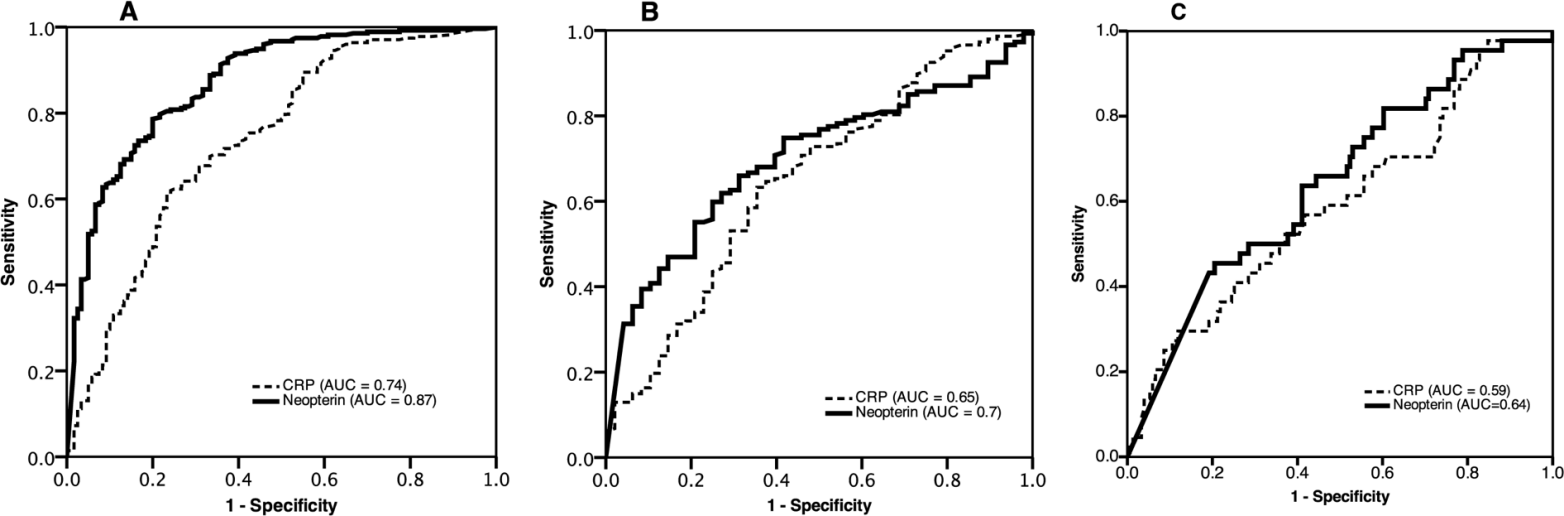
ROC curves showing AUC for neopterin and CRP for prediction of CD4 cell strata using three different cut-off levels: <500 cells/mm^3^, <350 cells/mm^3^, <100 cells/mm^3^. A: CD4 cell count <500 cells/mm^3^. Includes all participants. B: CD4 cell count <350 cells/mm^3^. Includes HIV+ patients. C: CD4 cell count <100 cells/mm^3^. Includes HIV+ patients.

To investigate the predictive value of neopterin and CRP plasma levels for CD4 cell count, AUC for these markers were analyzed at pre-defined CD4 threshold levels. The AUC for neopterin as a marker for CD4 cell count <500 cells/mm^3^ for all included patients was 0.87. For identification of HIV+/TB+ subjects with CD4 cell count <350 cells/mm^3^ and <100 cells/mm^3^ the respective figures were 0.7 and 0.64. CRP showed weak correlation to CD4 cell count at all examined levels in HIV+/TB+ subjects, with poor capacity to identify patients with CD4 count <350 cells/mm^3^ (AUC = 0.65) and <100 cells/mm^3^ (AUC = 0.59, Fig 3).

## Discussion

We examined plasma levels of two biomarkers related to immune activation and inflammation, neopterin and CRP, in subjects with TB, and correlated the levels of these markers to CD4 cell count before and after treatment initiation. Both neopterin and CRP levels were elevated in TB patients as compared to those in healthy controls; in particular, neopterin levels were high in HIV/TB-coinfected persons. Furthermore, neopterin levels showed significant inverse correlation with CD4 cell count, both in HIV+ and HIV-negative individuals. These findings support the role of immune activation in TB pathogenesis [15], and suggest that immune activation may be involved in TB-related CD4 lymphocytopenia.

Immune activation involves multiple pathways of the immune system, and can be assessed by several methods. In TB, the activation of macrophages for intracellular killing is essential. We chose to use neopterin to measure the degree of immune activation as this molecule (reflecting IFN-gamma secretion by macrophages upon activation) is a well-established marker of immune activation, and has high predictive capacity for HIV disease progression [16,17]. Secondly, rapid tests for semi-quantitative neopterin testing are available [18], which potentially could allow for its use as an alternative method to CD4 cell analysis at peripheral levels of the health care system in resource-limited settings.

To our knowledge, this study presents the hitherto largest material of data on neopterin in TB-patients with or without HIV coinfection (195 TB+/HIV-; 170 TB+/HIV+). Median neopterin levels in our participants (54 vs. 23 nmol/l, with and without HIV coinfection, respectively) are similar to those reported in other studies conducted in India, Zimbabwe and USA (sum of participants 126 TB+/HIV-; 102 TB+/HIV+; [19–23]). In accordance with our study, Immanuel et al found higher neopterin levels in HIV/TB coinfected subjects with CD4 cell counts <200 cells/mm^3^ [21]. However, we also observed a correlation between neopterin levels and CD4 cell counts in HIV-negative TB patients.

Since subnormal CD4 cell counts have been reported in apparently healthy HIV-negative individuals from different countries in sub-Saharan Africa (including the region where our study was conducted [24]), we also analyzed CD4 cell count, neopterin and CRP in healthy controls. However, these subjects had levels within normal range, implying that the abnormal levels found are not related to background population factors.

Mild but persistent elevations of neopterin are common in HIV-positive persons not receiving ART [17,25]. Ayehunie et al compared neopterin levels in HIV-positive subjects from Ethiopia and Sweden, and found significantly higher levels in Ethiopians for unknown reasons [26]. Since TB is a common OI in Ethiopia, which may be missed in routine clinical investigations [27], it is possible that unrecognized TB could explain the elevated neopterin levels among some participants in that study.

The degree of IA was thus highest in persons with HIV/TB coinfection, although it was also clearly elevated in HIV-/TB patients. The increased immune activation seen in TB-patients can have several explanations. Activation of macrophages likely occurs at the site(s) of infection as a result of tissue migration of peripheral CD4 T-cells. Consequently, this would lead both to decreased levels of CD4 cells in peripheral blood as well as increased macrophage activation. In support of this mechanism, we found that neopterin levels correlated inversely with CD4 cell count, and this was interestingly observed both in HIV+ patients and in HIV-negative TB cases with low CD4 cell count.

Furthermore, we found that high neopterin levels at ATT initiation was correlated to increasing CD4 cell count at TB treatment completion in HIV-negative persons. A similar non-significant trend was observed in HIV-positive individuals (not on ART). One possible explanation for this finding could be that adequate control of the underlying cause of immune activation–TB–would allow for restoration of CD4 cell homeostasis. However, our study design did not permit us to draw any causal conclusion for this correlation, nor to assess the influence of other aspects of TB pathogenesis on CD4 cell evolution. Since we were unable to analyze neopterin levels in plasma during follow-up we could not determine whether increasing CD4 cell count after ATT was correlated to decreasing neopterin concentrations. Furthermore, although all participants in this analysis completed ATT we could not exactly determine whether other conditions may have occurred during this period that could have affected CD4 cell evolution.

The increased levels of immune activation found in TB patients, and the correlation with CD4 cell counts, suggest that TB could be a contributing factor to HIV disease progression. Although such an association has been proposed previously, data on this issue are conflicting. Whereas in vitro experiments show increased HIV replication in TB-infected macrophages [28], epidemiological studies do not support that TB leads to a more rapid course of HIV disease [29].

In addition to neopterin we also measured levels of CRP, using a highly sensitive assay. CRP is an acute-phase reactant, mainly reflecting the level of systemic inflammation. Similar to neopterin, mild elevations of CRP are common in HIV infection. CRP has been suggested for use as a biomarker for identification of HIV-positive subjects at high likelihood of TB [30], but its relation to CD4 cell count has not been investigated. Although TB patients exhibited raised levels of CRP, we did not find any significant difference with regard to HIV coinfection status, and the correlation with CD4 cell count was weak.

Since both neopterin and CRP can be measured using rapid tests we also aimed to evaluate the performance of these markers as alternative methods to CD4 cell analysis for estimating immunosuppression. Despite considerable deployment of CD4 cell machines in low-income countries this methodology will be difficult to introduce at all levels of the health care system where PLHIV are treated.

However, the performance of neopterin in this regard was poor. CRP had even lower predictive capacity for severe immunosuppression, approaching chance (0.5) for this threshold [14]. Indeed, a previously constructed algorithm based on symptoms and clinical findings to predict severe immunosuppression (CD4 cell count <100 cells/mm^3^) had a better AUC than either neopterin or CRP based on the same patient material [11] (0.75 vs 0.64 and 0.58, respectively), S2 Fig. Since CRP did not show any clear correlation with CD4 cell count in our population, it is unlikely that the combination of these two markers would increase the predictive performance and allow for use as an alternative method for immunosuppression.

This study was performed in patients seeking care at health centers in Ethiopia, a representative setting for where most people coinfected with HIV and TB receive care globally. This also entails a limitation with regard to TB diagnosis, which was based on routinely available methods in Ethiopian health facilities. Thus microbiological confirmation was restricted to sputum smear microscopy. Although neopterin is a well-established marker for immune activation, a combination of soluble and cellular biomarkers associated with immune activation (such as TNF-α, proportion of HLA DR-positive lymphocytes, β2 microglobulin, and soluble CD14 receptor) might have given a more detailed description of immune activation. Finally, we did not have access to follow-up plasma samples for investigation of the evolution of biomarker plasma levels, nor a control group of HIV+ patients without TB.

In conclusion, we found enhanced immune activation in TB patients, with significantly higher levels of neopterin in HIV-coinfected individuals. High neopterin levels were inversely correlated to low CD4 cell count in TB patients, irrespective of HIV serostatus. This implies that immune activation is involved in TB-related CD4 lymphocytopenia, and suggests a possible role of TB in HIV disease progression through this mechanism.

## Supporting Information

S1 Fig Scatterplot showing the relation between neopterin levels and CD4 cell count at baseline for TB patients stratified by HIV serostatus (n = 365). Spearman rank correlation for **A**: HIV+/TB patients was -0.35 (p<0.001) and for **B:** HIV-/TB patients -0.51 (p<0.001). The upper cut-off level of neopterin was 111nmol/l according to the specified detection limit (as specified by the manufacturer).
(TIF)

S2 Fig Predictive capacity of neopterin and CRP for prediction of CD4 cell strata <100 cells/mm^3^ compared to a previously constructed clinical algorithm [11]. (TIFF)
