# Supplementary figures and images for: Plasma Levels of Neopterin and C-Reactive Protein (CRP) in Tuberculosis (TB) with and without HIV Coinfection in Relation to CD4 Cell Count
Source: PLoS One. 2015 Dec 2;10(12):e0144292. doi: 10.1371/journal.pone.0144292 (PMC4668010; doi:10.1371/journal.pone.0144292)

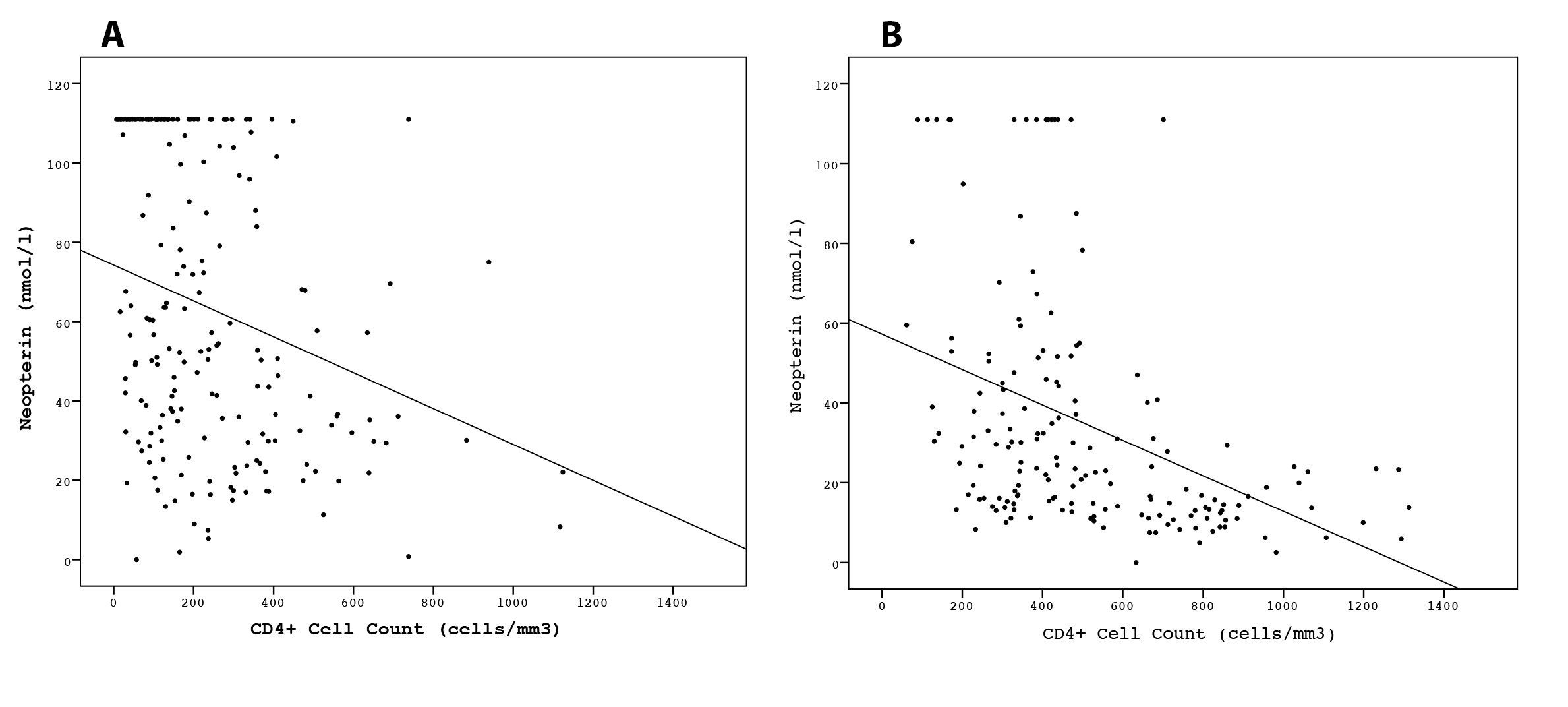

Supplement: S1 Fig — Spearman rank correlation for A: HIV+/TB patients was -0.35 (p<0.001) and for B: HIV-/TB patients -0.51 (p<0.001). The upper cut-off level of neopterin was 111nmol/l according to the specified detection limit (as specified by the manufacturer). (TIF) [file pone.0144292.s001.tif]

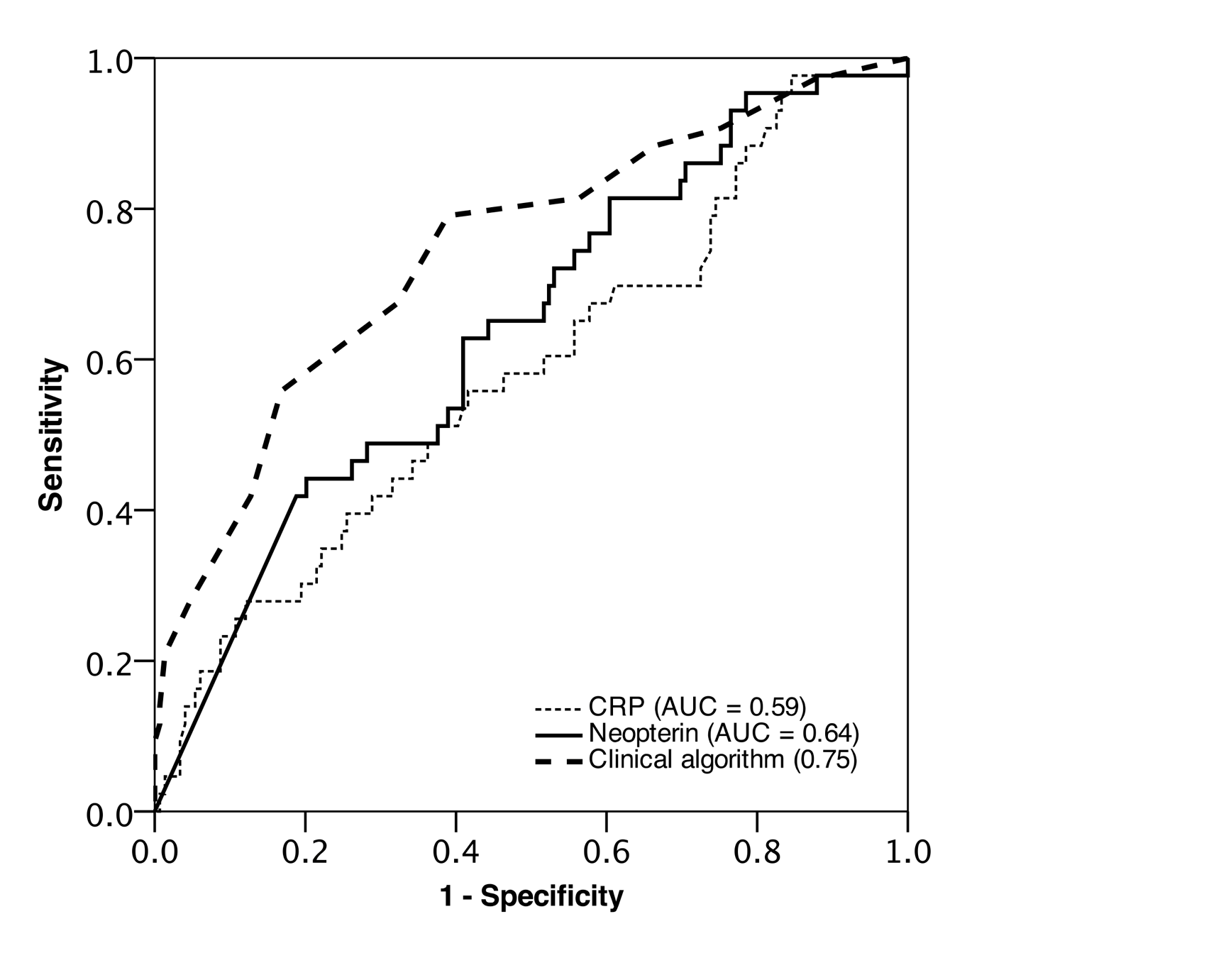

Supplement: S2 Fig — (TIFF) [file pone.0144292.s002.tiff]
